# Regional Differences in the Accumulation of SNPs on the Male-Specific Portion of the Human Y Chromosome Replicate Autosomal Patterns: Implications for Genetic Dating

**DOI:** 10.1371/journal.pone.0134646

**Published:** 2015-07-30

**Authors:** Beniamino Trombetta, Eugenia D'Atanasio, Andrea Massaia, Natalie M. Myres, Rosaria Scozzari, Fulvio Cruciani, Andrea Novelletto

**Affiliations:** 1 Dipartimento di Biologia e Biotecnologie “C. Darwin”, Sapienza Università di Roma, Rome, Italy; 2 AncestryDNA, Provo, Utah, United States of America; 3 Istituto di Biologia e Patologia Molecolari, Consiglio Nazionale delle Ricerche, Rome, Italy; 4 Dipartimento di Biologia, Università di Roma “Tor Vergata”, Rome, Italy; University of Perugia, ITALY

## Abstract

Factors affecting the rate and pattern of the mutational process are being identified for human autosomes, but the same relationships for the male specific portion of the Y chromosome (MSY) are not established. We considered 3,390 mutations occurring in 19 sequence bins identified by sequencing 1.5 Mb of the MSY from each of 104 present-day chromosomes. The occurrence of mutations was not proportional to the amount of sequenced bases in each bin, with a 2-fold variation. The regression of the number of mutations per unit sequence against a number of indicators of the genomic features of each bin, revealed the same fundamental patterns as in the autosomes. By considering the sequences of the same region from two precisely dated ancient specimens, we obtained a calibrated region-specific substitution rate of 0.716 × 10^-9^/site/year. Despite its lack of recombination and other peculiar features, the MSY then resembles the autosomes in displaying a marked regional heterogeneity of the mutation rate. An immediate implication is that a given figure for the substitution rate only makes sense if bound to a specific DNA region. By strictly applying this principle we obtained an unbiased estimate of the antiquity of lineages relevant to the genetic history of the human Y chromosome. In particular, the two deepest nodes of the tree highlight the survival, in Central-Western Africa, of lineages whose coalescence (291 ky, 95% C.I. 253–343) predates the emergence of anatomically modern features in the fossil record.

## Introduction

The uniparental genetic systems have been so far consideredthe clearest markers of past human dispersals, since they allow the reconstruction and dating of molecular radiations on the basis of parsimony [1].

Among the two uniparentally inherited portions of the genome, mitochondrial DNA and the male-specific portion of the Y chromosome (MSY), the latter has been so far too large to be scanned for variation among individuals to completion. High throughput methods have been applied for the discovery of large arrays of polymorphic markers with PCR-based methods (see the pioneering work of Underhill et al. [2]). Though these, too, were addressed to regions with low-to-null homology with the X chromosome, they relied on the availability of MSY genomic sequence at the time.

Since next generation sequencing (NGS) technologies have become available, a flood of data is emerging for variation at nucleotide level along the MSY. Different methods have been applied in recent works [3–10],which provide variable accessibility to different subregions of the chromosome, but all supposedly detect variants in an unbiased way within their respective targeted DNA segments. The results of different studies can be merged by working out a minimal DNA region shared across them, with two main advantages: 1) the presence of each derived variant allele shared by at least two subjects can be cross-checked and 2) a much larger spectrum of lineages can be assembled, allowing the description of the accumulation of variants in a sample of chromosomes as representative of the whole male population as possible. In this context, the composition of the merged datasets further benefits of the inclusion of rare lineages, that might be represented in some studies but not in others, e.g haplogroups (Hg) A-00, D and A0 [4, 5, 11].

Once the distribution of variants can be regarded as reporting their true emergence and persistence in the population, fundamental questions on the density of variant sites, on mutational processes at the basis of polymorphism and their fluctuations along the chromosome can be approached. The answers are of crucial importance when attempting to arrive at a figure for the substitution rate to be used in the absolute genetic dating of lineages currently observed or retrieved from fossil material by applying a molecular clock concept under strict neutrality. In fact, this requires that mutation and substitution rates could be equated. The recent availability of autosomal, genome-wide estimates of the *de novo* mutation rate [12–14] prompted some authors [4, 11] to adopt it as a base to calculate a substitution rate for the Y-chromosome, with due adjustments.This operation is not free of assumptions (thoroughly discussed in the Supplemental text of Scozzari et al.[4]) and is currently a subject of vivid debate [15–17]. Specifically, there are several unknowns. The first is whether particular genomic features affect the mutational process at the origin of new variants. The second is whether, once appeared, the chance of survival is evenly distributed across nucleotide positions, or if some type of selection (most likely purifying) is operating or has operated pervasively on portions of the Y chromosome. Indeed, a very recent report [18] showed that the pedigree-based measure of the MSY point mutation rate is higher than that obtained by transposing the autosomal rate. Also, the same report unveils remarkable variation of the rate at the multi-megabase scale, i.e. among four MSY regions which emerged from different DNA-rearrangement events during long term evolution of the chromosome.

Factors affecting the rate and pattern of the mutational process are being identified for autosomal DNA [19, 20], but the validity of the same relationships for the MSY cannot be taken for granted. In fact the region is free of allelic recombination, a factor repeatedly shown to co-vary with the nucleotide substitution rate [21–23]. Additionally the MSY is enriched in intra- and inter-chromosomal segmental duplications, and displays extensive structural rearrangements even in comparison with our closest living relatives, the great apes [24]. This limits the applicability of inter-specific comparisons, commonly used for the autosomes since the seminal work by Wolfe et al. [25].

In this work, we introduce the use of a robust intra-specific phylogeny to test the regional evenness of the occurrence of mutations and the possible correlates of this phenomenon in a 1.5 Mb portion of the MSY. We explore the distributional pattern of thousands of derived alleles that have persisted in the human population for as much as 300,000 years under widely different environmental conditions. In this way we use the phylogeny as a surrogate for a direct observational study of mutational events in the MSY that could not be otherwise performed. We show significant variations in the occurrence of variable positions at the multi-kilobase scale, with some degree of variation across lineages and time windows represented in the tree. Of utmost importance is that inferences restricted to the diversity of specific subregions may lead to widely discrepant results, replicating previous observations from the X chromosome [26]. The use of an appropriate sizing of the experiment and tuning of the parameters used in dating then becomes an essential step to arrive at figures reliable for putting upper and lower time limits to specific population processes. In particular, our findings highlight the need to match high precision (i.e. small fluctuations due to the large number of mutations revealed by NGS) with high accuracy (i.e. fluctuations with reduced systematic bias, due to the use of a rate calibrated for the re-sequenced region) in the estimation of node ages.

## Materials and Methods

### The samples

We report here the results obtained in 104 subjects coming from three different datasets: (1) 68 subjects examined in the same experiment of, but not described in detail in ref.[4]; (2) 9 additional males carrying haplogroups relevant for reconstructing the Y-chromosomal phylogeny and sequenced in the present study; (3) 27 unrelated males from the diversity panel of the Complete Genomics company. The affiliation to Y chromosome haplogroup and geographic provenance of all subjects are described in S1 Table.

Part of the DNA samples in datasets 1 and 2 were obtained from the Coriell Institute Cell Repository (Camden, New Jersey, USA), the National Laboratory for the Genetics of Israeli Populations (Tel Aviv, Israel), and the Sorenson Molecular Genealogy Foundation (Salt Lake City, Utah), and used in accordance with the conditions specified by the above Institutions. The remaining samples in datasets 1 and 2 are from collections of the authors, assembled in the 1980's, 1990's and 2000's. The original sampling was performed by colleagues and operators at a number of collaborating Institutions. As far as the proposed research did not involve any issue relevant for the donor's health, only a subset of the WMA Declaration of Helsinki and COE Oviedo Convention prescriptions were applicable and obeyed. For these reasons written consent was requested in most cases but, in some series collected before 1995, oral consent was considered sufficient and simply recorded in the corresponding log sheets (filed at the collecting Institutions). In all cases the consent included also storage and future use of the sample. Anonymized blood or DNA samples were then received at the authors' laboratories. The study was prospectively examined and approved by the "Policlinico Umberto I, Sapienza Università di Roma" ethical committee (document number 496/13), who expressly considered the list of collaborators, anonymity of samples and the compliance with consent regulations of previous publications which included the same samples.

### Sequencing, data quality control and data filtering

For the 9 subjects sequenced in this study, DNA quality control, library preparation, sequencing and data filtering were as for the 68 samples of dataset (1)[4]. Overall 1,495,512 bases of the target region, subdivided into 5,274 baited fragments, were resequenced.

Genotyping results (VCF files) for the genomes of the 27 subjects of dataset (3) were downloaded from the FTP site of the Complete Genomics company (ftp://ftp2.completegenomics.com/vcf_files/Build37_2.0.0/). For each VCF file, we extracted single nucleotide substitutions falling within the same 1,495,512 bases mentioned above. For each sample, low quality mutations (FT = VQLow) and/or mutations with allelic depth ≤ 2 were discarded.

After a final check for phylogenetic consistency using all the filtered positions, we removed 3 sites (nucleotide positions: 6670461, 8417317, 19044813), which were recurrent in 4–6 branches of the tree. Two positions (19319427 and 19431990) reported as variant in subject NA12889 but not in his male descendants [5] were retained.

We downloaded the alignment.bam files of Ust’-Ishim [27] and Loschbour [28] genomes from the European Nucleotide Archive (ENA) website under the accession numbers PRJEB6622 and PRJEB6272, respectively. We used samtools and bcftools (versions 1.1) to obtain the.vcf files for the variant positions of the Y chromosome of both genomes. The variants used for subsequent analyses were filtered in two steps: 1) only positions within the 1.5 Mb region sequenced in Scozzari et al. [4] were considered and 2) only variants with an “alternative” allele depth higher than the “reference” allele depth were retained. The mean depth for the 1.5 Mb region extracted from the Ust’-Ishim Y chromosome was about 22X, with only 42 bases having depth <2.For the same 1.5 Mb region extracted from the Loschbour genome,the mean depth was about 11X, with only 3554 bases having depth <2.

The 1.5 Mb of the Y chromosome analyzed were partitioned in 19 bins of consecutive fragments, each approaching 80 kb (with the exception of the small region chrY:6655516–6673365) of effectively covered bases (col. 7 in Table 1). The number of mutations falling in each bin was calculated and was normalized per 100 kb of effectively sequenced bases.

**Table 1:** Features of the 5 genomic regions considered in this work, subdivided into 19 bins.

Region	Bin n.	Initial pos.^1^	Final pos.^1^	Genomic span	N. of baited fragments	Effectively sequenced bases	Gene content^2^	CpG content^3^	Replication time score (BG02 cell line)
A	1	2690918	2910156	219239	285	91454	6.20	1318	1.14
B	2	6655517	6673365	17849	33	8275	0	70	-0.30
C^4^	3	7540768	7671720	130953	289	79812	0.56	1028	-0.77
	4	7671760	7945341	273582	394	79655	0.31	584	-0.87
	5	7946953	8156861	209909	457	79642	0	1004	-0.71
	6	8156863	8382932	226070	397	79674	0	660	-0.72
	7	8383002	8489128	106127	186	78966	0	1090	-0.93
	8	8489176	8595654	106479	147	79820	0.62	1222	-0.78
	9	8595830	8739563	143734	257	80846	0.77	832	-0.49
D	10	14629906	14887780	257875	256	83305	6.64	1184	1.28
	11	14888375	15056945	168571	218	83295	13.54	1090	1.46
	12	15056995	15436522	379528	332	82726	3.52	710	1.21
	13	15436558	15727001	290444	282	83244	5.42	952	0.18
	14	15730403	15957784	227382	291	83106	2.00	1292	-0.16
E	15	18553309	18714071	160763	246	84781	0	614	-0.62
	16	18714094	18912763	198670	292	84043	0	550	-0.56
	17	18912843	19148792	235950	330	84363	0	634	-0.66
	18	19148988	19343219	194232	309	83940	0	622	-0.85
	19	19343595	19549929	206335	273	84565	0	638	-0.68

1. GRCh37/hg19 coordinates

2. as % bases overlapping UCSC genes in the sequenced fragments

3. as positions residing in CpG's in the sequenced fragments

4. classified as non-palindromic ampliconic in ref. [18] with no highly similar paralogues on the Y chromosome

### Phylogenetic analysis and dating

Parsimony analysis of the merged dataset was performed with MEGA [29] and NETWORK [30]. The.out file produced by this latter program was manipulated in a spreadsheet to directly obtain the assignment of mutations to each of the tree branches.

Absolute dating of tree nodes was obtained with BEAST [31]. The input file consisted of the matrix of the individuals' genotypes at the 3,373 variable positions plus 3 and 8 for private variants of the Ust'-Ishim and Loschbour specimens, respectively. These were given sampling dates of 45,000 and 7,205 years ago, respectively. An initial run was used to obtain a seeding tree to be used in all subsequent runs. In all runs we used a GTR model for nucleotide substitutions under a strict clock. An expansion model was used for the population size, to appropriately account for the faster recent growth, and rather flat priors were used, i.e. lognormal[10,3] for the current population size, exp[0.2] for the ancestral/current population size ratio and uniform[0, 0.00133] for the growth rate/year in the expansion phase, using the upper bound reported in the literature for pre-agricultural cultures [32–34]. We used runs of 10 million steps each, sampled every 10,000 steps. The initial 20% of each run was discarded as burn-in and the outputs analyzed with Tracer and Tree Annotator.

Heterogeneity χ^2^and regression tests were performed with R, always excluding the small bin n.2 (18 points).

### Extracting information on genomic features

Information on the genomic features here considered was retrieved by using the UCSC Table Browser (http://genome.ucsc.edu/cgi-bin/hgGateway). In particular, the list of the genomic coordinates for the 5,274 baited fragments was intersected with tracks for UCSC genes, transcribed regions, EST's and GC content. The intersection was basewise, to measure the precise percentage of base positions overlapping the above tracks. For GC content and simple repeats we repeated the procedure using, instead, the genomic coordinates (beginning and end) of the 19 bins.

The replication time scores derived from two Embryonic Stem Cells (ESCs) datasets (BG01 and BG02; Chip ID: BG01_UGA_15473002&15707202CGH and 293676i&294988ii_BG02hESC_ave) were downloaded from http://www.replicationdomain.com [35]. Positive values were indicative of early replication and negative values were indicative of replication later during S-phase. The score assigned to each bin was the average of all values extracted for that bin.

## Results

### Assembling a set of mutations

A total of 104 male subjects were successfully genotyped at 1,495,512 DNA positions from 5 Y-chromosomal regions (A-E in Table 1) selected to show low-to-null homology with the X chromosome[36, 37] (see Figure 1 in ref. [4]).

The sequencing results derive from three independent series of subjects and two different analytical procedures (see Materials and Methods).

**Fig 1:**
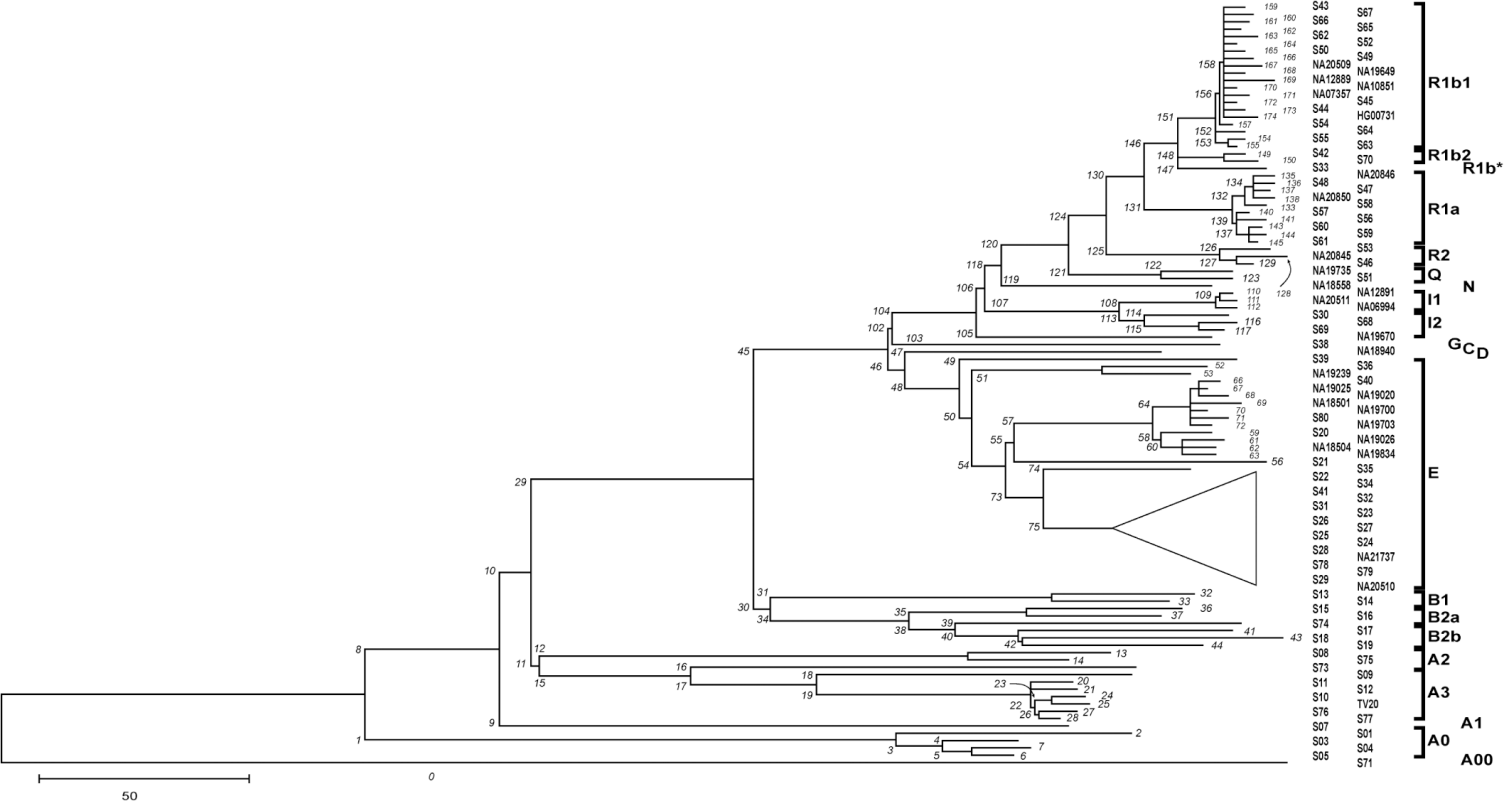
Maximum parsimony tree showing the phyletic relationships of 104 chromosomes. The individual Id's (as in S1 Table) are reported on the right, aligned with the corresponding branch. Clades corresponding to major haplogroups are bracketed or indicated individually at the far right, following the nomenclature of van Oven et al. [64](note the difference with ref. [4]). Branches are numbered (in italics) and mutations assigned to them are listed in S2 Table. Note that the tree is unrooted and variants defining branch 0 are identified solely as different from the reference sequence. The clade corresponding to Hg E1b1b-M35 has been collapsed since it is discussed in detail in a dedicated paper [65].

An unrooted maximum parsimony tree based on the observed 3,373 variable sites is depicted in Fig 1. In general, this tree recapitulates the known Y-chromosomal phylogeny. Features related to the particular composition of our sample of chromosomes, not present or visible in the previously published version [4] and not appreciable in other works are highlighted in the S1 Text.

The parsimony reconstruction inferred 17 (0.50%) positions at each of which 2 independent events generated the same (11) or different (6) derived alleles on different phylogenetic backgrounds, hereafter referred to as recurrent and double hit mutations, respectively (S2 Table, col. F). The significantly lower proportion of positions suffering multiple events as compared to a recent study (0.92%) [9] can be at least partly explained by the larger number of chromosomes examined therein. Among the 3,373 variable positions, 368 were polymorphic only in the Complete Genomics (CG) subset, 2,664 among the remaining subjects, and 347 in both datasets. The number of positions previously known to be variable and recorded in dbSNP(138) (643/3,373) accounted for a higher percentage (79.5%) among mutations shared by ours and CG's datasets than those found in CG only (23.9%) or in our dataset only (10.5%). This depends on the more complete representation of the worldwide diversity in the pooled set, coupled with the higher chance of having captured variation in deep branches in previous studies.

When standardized to the amount of sequence effectively analyzed, we obtained average densities of 225.5 variable positions and 226.7 mutations every 100 kb.

### Heterogeneous occurrence of mutations in different genomic blocks and their implications for dating

We first wanted to specifically test the evenness of the occurrence of mutational events between the 5 genomic regions here considered (Table 1). We found a strongly heterogeneous accumulation of variants (χ^2^ = 32.7, df = 4, p = 1.4 × 10^−6^), with more than 230 variants/100 kb in regions C and E but less than 200 variants/100 kb in regions A, B and D. We then proceeded in dissecting this variation. To this aim we subdivided the four largest regions (A, C, D, E, Col. 1, Table 1) in bins, each of approximately 80 kb of effectively sequenced bases, because: 1) the size of all regions approaches an integer multiple of this value; 2) this value could be easily approached by summing up the length of subsequent baited fragments; 3) this value is large enough to ensure that the number of mutations falling within each bin is large, to reduce stochastic fluctuations. The results are reported in Table 2. Over the whole tree, the number of mutational events ranged 150 to 279 per bin (excluding the small bin n. 2), with values that were far from proportionality to the amount of sequenced bases (χ^2^ = 115.5, df = 17, p = 2.2 × 10^−16^). After standardization per sequenced bases, this discrepancy further increased, reaching a two-fold variation (from 181 to 353 mutations/100 kb). Taken at face value, this finding is compatible with both differential propensity to mutational events and/or differential viability of derived alleles in the phylogeny across bins. At any rate, it shows that assuming a uniform substitution rate for different chromosomal regions is simplistic.

**Table 2:** Distribution of mutational events in 19 bins in total.

Region	Bin n.	Effectively sequenced bases	Whole tree (all positions)				Whole tree (CpG's)				Whole tree (non-CpG's)			
			Abs.	/100kb	Tr	Tv	Abs.	/100kb^1^	Tr	Tv	Abs.	/100kb	Tr	Tv
A	1	91454	178	194.6	111	67	20	1441.2	19	1	158	175.3	92	66
B	2	8275	15	181.3	8	7	2	845.9	2	0	13	158.4	6	7
C	3	79812	199	249.3	126	73	27	1288.0	26	1	172	218.3	100	72
	4	79655	178	223.5	108	70	20	733.2	17	3	158	199.8	91	67
	5	79642	147	184.6	91	56	13	1260.6	11	2	134	170.4	80	54
	6	79674	185	232.2	105	80	18	828.4	16	2	167	211.4	89	78
	7	78966	279	353.3	172	107	55	1380.3	53	2	224	287.6	119	105
	8	79820	201	251.8	141	60	29	1530.9	28	1	172	218.8	113	59
	9	80846	196	242.4	123	73	16	1029.1	16	0	180	225.0	107	73
D	10	83305	165	198.1	114	51	19	1421.3	17	2	146	177.8	97	49
	11	83295	157	188.5	98	59	17	1308.6	16	1	140	170.3	82	58
	12	82726	150	181.3	92	58	16	858.3	16	0	134	163.4	76	58
	13	83244	181	217.4	121	60	20	1143.6	18	2	161	195.6	103	58
	14	83106	169	203.4	103	66	15	1554.6	13	2	154	188.2	90	64
E	15	84781	172	202.9	106	66	17	724.2	15	2	155	184.2	91	64
	16	84043	164	195.1	109	55	19	654.4	18	1	145	173.7	91	54
	17	84363	252	298.7	153	99	30	751.5	28	2	222	265.1	125	97
	18	83940	202	240.6	133	69	22	741.0	20	2	180	216.0	113	67
	19	84565	200	236.5	131	69	21	754.4	18	3	179	213.3	113	66
	Total	1495512	3390		2145	1245	396		367	29	2994		1778	1216
	χ2 (17 d.f.)		115.6		73.0	58.2	76.3		80.4	n.t.	75.8		37.1	58.2
	P		2.20×10^−16^		6.63×10^−9^	2.07×10^−6^	1.75×10^−9^		3.31×10^−10^		2.14×10^−9^		0.003	2.05×10^−6^

1. Mutations at CpG dinucleotides every 100 kb of positions in CpG dinucleotides.

This strongly heterogeneous regional distribution of mutation events predicts that using a phylogeny based on sites drawn from regions with high or low density of variable positions, and applying to them the same substitution rate, will result in grossly over- and under-estimated node ages, respectively. We exemplify this by dating the trees obtained from positions residing in the two regions with the highest and lowest accumulation of variants (S1 Fig). While the tree topologies undergo slight changes, due to the reduced number of informative sites, the age of comparable nodes is drastically moved to the past in the case of the region with an increased occurrence of mutations (S1A Fig) as compared to the other one (S1B Fig). A discussion of the implications of these time shifts is given in S2 Text.

### Heterogeneity by mutational type and site

We explored the occurrence of mutations in non-CpG and CpG dinucleotides, the latter known to undergo transitional mutations at high rate. Along the whole tree, 2,994 mutations occurred in non-CpG positions, with a density ranging between 173 and 287/100 kb (χ^2^ = 75.8, df = 17, p = 2.1 × 10^−9^). On the other hand, 396 mutations occurred at the C or G position in CpG dinucleotides (Table 2), which comprehensively account for 16,094 bp in the reference sequence. This corresponded to 2,460.5 mutations per 100 kb of positions in CpG's, a figure 12.2 fold higher than in non-CpG positions. Also in this case the distribution of mutations at CpG sites across the 19 bins was not proportional to the amount of sequence obtained in each bin (χ^2^ = 76.3, df = 17, p = 1.7 × 10^−9^, Table 2). Overall, we obtained a strong and highly significant (r = 0. 76, p = 0.0003) covariation between the number of mutations at non-CpG per unit sequence and the number of mutations at CpG per available CpG (S1 Fig), showing that the scaling-up of the substitution rate at CpG's is of similar magnitude across bins.

At non-CpG and CpG sites, transitions outnumbered transversions of factors 1.5 and 12.7, respectively. For non-CpG sites, transversions displayed larger variations across bins than transitions (coefficients of variation = 0.23 and 0.15, respectively).

As to coding variants, when standardized to the total exonic sequence represented in our fragments (33,377 bp), coding variants (76) had a density (227.7/100 kb) comparable to the entire dataset.

### Patterns of occurrence of mutations through lineages and time

In order to gain insights on the factors possibly affecting the uneven distribution of mutations across bins, we sectioned the tree both horizontally (considering different haplogroups transmitted through ages) and vertically (considering all haplogroups within different time windows).

When specific haplogroups were considered, we observed six highly significant (p-values range = 0.002–0.027) and two borderline (p < 0.08) departures from proportionality between the number of mutations and the amount of sequence analyzed (Table 3). The excess of mutations in the region chrY: 8383002–8489128 (bin 7) was reproducible in all haplogroups, whereas the excess in the region chrY: 18912843–19148792 (bin 17) was reproducible in all haplogroups except A0 and A2'3.

**Table 3:** Distribution of mutational events in 19 bins, by haplogroup.

Reg.	Bin n.	Selected haplogroups^1^ (all mutations)															
		A00		A0		A1		A2'3		B		DE		CF^2^		R1	
		Abs.	/100kb	Abs.	/100kb	Abs.	/100kb	Abs.	/100kb	Abs.	/100kb	Abs.	/100kb	Abs.	/100kb	Abs.	/100kb
A	1	18	19.7	16	17.5	9	9.8	27	29.5	30	32.8	43	47.0	28	30.6	3	3.3
B	2	1	12.1	0	0.0	0	0.0	6	72.5	0	0.0	5	60.4	2	24.2	1	12.1
C	3	23	28.8	13	16.3	7	8.8	27	33.8	40	50.1	34	42.6	50	62.6	23	28.8
	4	20	25.1	15	18.8	12	15.1	21	26.4	27	33.9	35	43.9	39	49.0	13	16.3
	5	23	28.9	4	5.0	9	11.3	17	21.3	15	18.8	34	42.7	41	51.5	16	20.1
	6	18	22.6	10	12.6	5	6.3	24	30.1	33	41.4	46	57.7	43	54.0	15	18.8
	7	32	40.5	28	35.5	15	19.0	37	46.9	46	58.3	59	74.7	57	72.2	21	26.6
	8	31	38.8	21	26.3	8	10.0	23	28.8	24	30.1	46	57.6	41	51.4	14	17.5
	9	20	24.7	9	11.1	10	12.4	32	39.6	29	35.9	45	55.7	41	50.7	13	16.1
D	10	16	19.2	18	21.6	4	4.8	22	26.4	27	32.4	39	46.8	30	36.0	10	12.0
	11	13	15.6	12	14.4	7	8.4	33	39.6	22	26.4	31	37.2	37	44.4	15	18.0
	12	16	19.3	13	15.7	6	7.3	27	32.6	23	27.8	30	36.3	29	35.1	12	14.5
	13	12	14.4	13	15.6	3	3.6	30	36.0	29	34.8	45	54.1	43	51.7	10	12.0
	14	21	25.3	14	16.8	12	14.4	23	27.7	25	30.1	37	44.5	34	40.9	13	15.6
E	15	18	21.2	12	14.2	8	9.4	29	34.2	26	30.7	40	47.2	31	36.6	10	11.8
	16	23	27.4	14	16.7	2	2.4	23	27.4	25	29.7	34	40.5	40	47.6	12	14.3
	17	29	34.4	11	13.0	7	8.3	47	55.7	39	46.2	50	59.3	53	62.8	20	23.7
	18	26	31.0	13	15.5	5	6.0	33	39.3	27	32.2	35	41.7	54	64.3	22	26.2
	19	33	39.0	6	7.1	7	8.3	37	43.8	27	31.9	41	48.5	41	48.5	18	21.3
	Total^3^	393		242		136		518		514		729		734		261	
	χ2 (17 d.f.)	33.3		38.3		26.3		29.9		33.6		25.8		36.2		32.4	
	P	0.010		0.002		0.069		0.027		0.009		0.079		0.004		0.013	

1. Nomenclature as in ref. [64]

2. Includes R1

3. The sum across haplogroups is not 3390 as some Hg's are not shown and R1 is also considered in CF

We then asked whether this lack of proportionality followed the same pattern of highs and lows across haplogroups. No evidence for differences among 7 haplogroups (R1 excluded as already included in CF) for the pattern of heterogeneity was obtained by contingency χ^2^ (χ^2^ = 111.3, df = 102, p = 0.25).

**Fig 2:**
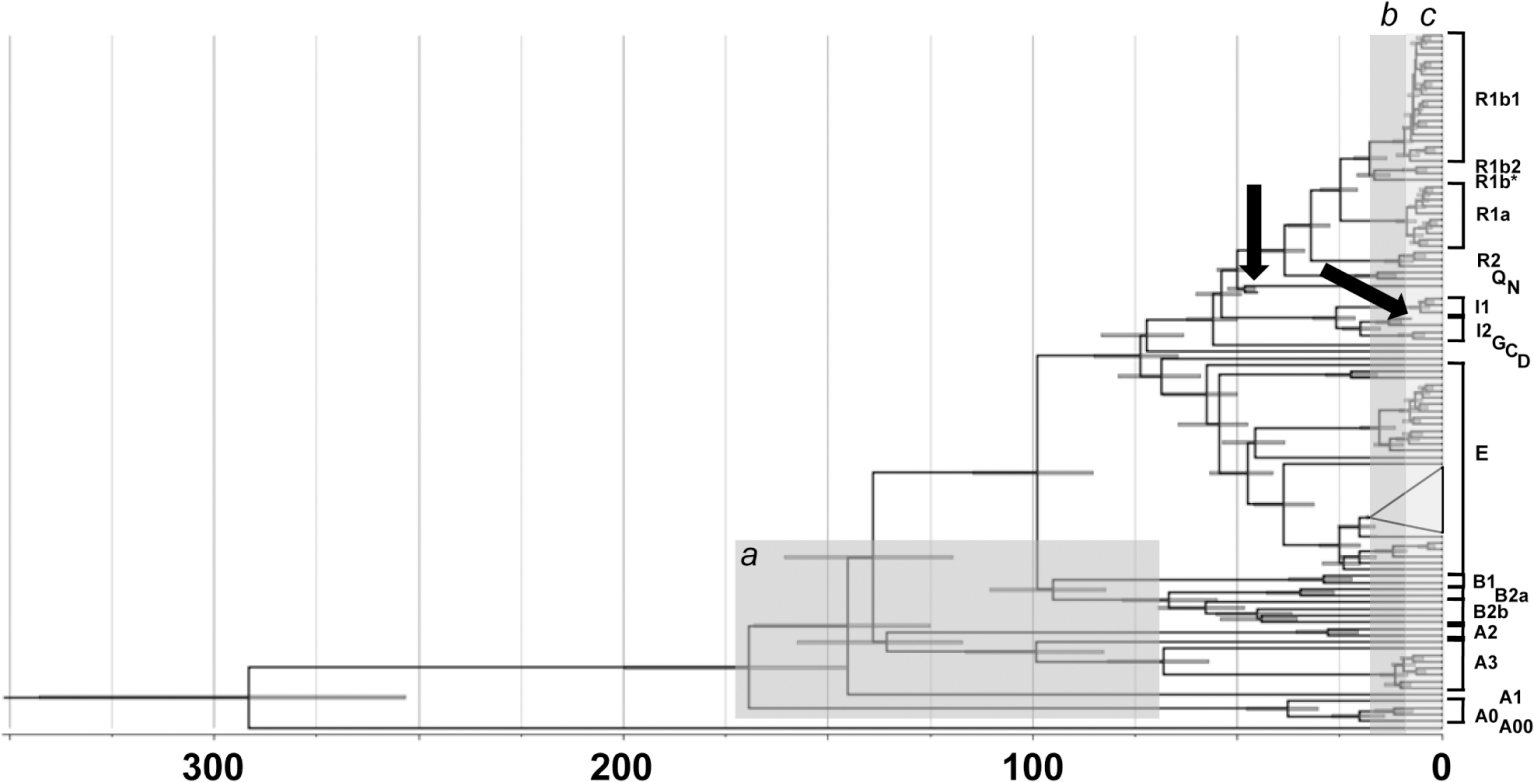
Dated tree including 104 subjects plus the Ust'-Ishim [27] and Loschbour [28] specimens (arrowed). These latter were used as calibration points, by means of normally distributed priors with means 45,000 and 7,205 years ago, respectively. The 95% C.I.'s for the age of each node are represented as grey bars. The clade corresponding to Hg E1b1b-M35 has been collapsed since it is discussed in detail in a dedicated paper [65]. Groups of branches discussed in the text and in Table 4 are shadowed: a) deep branches; b) terminal branches with rho < = 20; c) terminal branches with length < = 10 mutations. Note that the positioning of the root is the result of the Bayesian process and not of the assessment of ancestral/derived states in branch 0 based on an outgroup (e.g. the chimpanzee).

Then, we considered four classes of tree branches, corresponding to different time windows (greyed in Fig 2): very recent branches with a length of 10 mutations or less (approximately equivalent to the last 9.3 ky); recent branches defined as those that coalesce at nodes with an average distance from the tip (rho) of 20 mutations or less; deep African branches lying between the A0-T node and any node older than 68 kya; all other branches, lumped in a group of medium antiquity. Hg A00 was excluded as it crosses all ages and is represented by a single individual. Also in all of these partitions, the regions C and E (Table 1) showed the highest numbers of mutations/100 kb. Non-proportionality as compared to the amount of sequenced positions for each bin was replicated in the four time windows and reached significance for the deep (p = 0.003) and intermediate (p = 2.0 × 10^−11^) branches (Table 4).

**Table 4:** Distribution of mutational events in 19 bins, by time windows.

Reg.	Bin n.	Deep branches		Intermediate branches		Terminal branches			
						Rho < = 20		< = 10 mut.	
		Abs.	/100kb	Abs.	/100kb	Abs.	/100kb	Abs.	/100kb
A	1	16	17.5	111	121.4	32	35.0	10	10.9
B	2	1	12.1	8	96.7	4	48.3	1	12.1
C	3	14	17.5	126	157.9	38	47.6	19	23.8
	4	21	26.4	106	133.1	31	38.9	10	12.6
	5	9	11.3	79	99.2	36	45.2	19	23.9
	6	10	12.6	117	146.8	40	50.2	16	20.1
	7	36	45.6	172	217.8	41	51.9	21	26.6
	8	21	26.3	113	141.6	38	47.6	18	22.6
	9	17	21.0	117	144.7	42	52.0	24	29.7
D	10	19	22.8	102	122.4	28	33.6	10	12.0
	11	16	19.2	99	118.9	31	37.2	16	19.2
	12	12	14.5	82	99.1	38	45.9	24	29.0
	13	19	22.8	124	149.0	26	31.2	12	14.4
	14	16	19.3	88	105.9	44	52.9	23	27.7
E	15	17	20.1	105	123.8	30	35.4	18	21.2
	16	15	17.8	90	107.1	35	41.6	19	22.6
	17	24	28.4	148	175.4	47	55.7	19	22.5
	18	23	27.4	108	128.7	45	53.6	28	33.4
	19	17	20.1	111	131.3	38	44.9	23	27.2
	Total	323		2006		664		330	
	χ2 (17 d.f.)	37.2		87.2		18.9		26.8	
	P	0.003		2.0×10^−11^		0.331		0.061	

### Y-chromosomal patterns of occurrence of mutations as a function of genomic features

We regressed the number of mutations/100kb in each bin against a number of variables that may influence the occurrence of variations (S3 Table).

We considered three measures of the expression activity in our DNA segments, i.e. the overlap with exonic and transcribed sequences of UCSC genes, and with EST's. The results (S2 Fig) showed a weak decreasing trend of variable sites as the occupancy by exonic sequences, genes and EST's increased. However, in none of the cases the regression was significant (p-value range = 0.051–0.15). We noticed a highly variable amount of mutations/100 kb in bins with a very low overlap with transcribed sequences, indicating that additional features may add to the variation in the accumulation of variable sites in gene-free regions.

We also considered the GC content in each entire bin (S2 Fig) and over the sequenced segments of each bin. In these cases we observed strong positive correlations between the number of mutations/100 kb and the GC content in the whole tree (r = 0.63 and 0.55, p = 0.005 and 0.019, respectively).

We next analysed the occurrence of mutations as a function of the percentage of the genomic span of the bin occupied by simple repeats (S2 Fig). In this case we found a positive though not significant positive correlation for the whole tree (p = 0.09).

Finally, we looked at the occurrence of mutations/100 kb as a function of DNA replication time (S2 Fig). For both cell lines considered, we observed a relationship (r = -0.42, n.s. and -0.55, p = 0.018) over the whole tree, indicative of higher mutability of late replicating DNA segments, in agreement with previous studies on this relationship [38, 39]. Detailed results of the above analyses in 8 haplogroups are reported in S3 Table and commented in S3 Text.

Multiple regression against all 7 variables showed good predictability of the number of variants in 16 out of 18 bins (considering the exclusion of bin n. 2). Bins 7 and 17 displayed an higher than predicted density of variants, implying additional unexplored factors contributing to the polymorphism of these chromosomal segments (S3 Fig). In order to identify the most relevant variables among those here considered we applied a multiple stepwise regression analysis. The GC percent, amount of simple repeats and replication time were the best predictors for the density of all variants across bins (Multiple R = 0.786; p = 0.003) whereas GC percent and replication time were the best ones for the density of variants at non-CpG sites (Multiple R = 0.739: p = 0.003).

As to different time windows, the occurrence of mutations in bins among the very recent and recent branches was strongly negatively correlated with the content of expressed sequences (5 p-values ranging 0.034–0.003 out of 6 regressions). In the medium and deep branches the same trend was confirmed, with negative though not significant r values. In recent, medium and deep branches positive relationships were observed with GC and simple repeat content and the trend towards more mutations in late replicating DNA segments was confirmed.

### Calibrated dating of the tree nodes

In order to obtain estimates for the node ages in our tree, we took advantage of the data for the same region here examined extracted from the high coverage sequencing of two precisely dated ancient specimens [27, 28]. The dated tree is displayed in Fig 2 and the relevant aspect of its chronology discussed below. The substitution rate was estimated at 0.716 × 10^−9^/site/year (95% C.I. 0.619–0.815).

## Discussion

We present here a collection of mutational events in the MSY inferred from the phylogeny relating104 males re-sequenced at high coverage at about 1.5 Mb.

Our approach of targeted, deep sequencing (50×) ensures that the ascertainment of variants was unbiased over the surveyed DNA segments. These reside in 5 unique regions chosen to reduce to a minimum the inflation of the dataset with mutational events due to mechanisms related to the similarity with the X chromosome such as X-to-Y gene conversion [40–44]. The design of our baited fragments selected for sequences devoid of interspersed and simple repeat elements, eliminating uncertainties in the mapping of reads.

We show here that, despite these precautions, the occurrence of variable sites and inferred mutations in the Y-chromosomal phylogeny was quantitatively different along different chromosomal regions, reaching a twofold variation.

The main assumption in studies addressing the history of Y chromosomal diversity is that the markers utilized do not depart significantly from neutrality. Rozen et al. [45] used the straightforward approach of considering coding synonymous vs. non-synonymous substitutions, and interpreted the reduced diversity at non-synonymous sites as due to active purifying selection. The regional analysis performed here and the presence of exonic segments in some bins only did not allow the further subdivision of coding variants without substantial sampling fluctuations. Then, we used alternative approaches to assay the possible effects of selection in our dataset. We did not observe a reduction in the density of variable sites per unit of exonic sequence as compared to the rest of sequences. We indeed observed a depletion of variable sites in gene-containing bins but this seems to be a rather non-specific effect. In fact, while the presence of functional DNA segments imposes limits to the accumulation of variable sites, when the occupancy by genes is close to null, the amount of mutations varies more widely, replicating precisely the observations from the X chromosome [26]. Also, Wilson Sayres et al. [46] concluded that purifying selection on the Y chromosome acts mostly through linkage with sites in the ampliconic region, which was not directly examined here. Finally, we compared recent and deep branches of the tree, expecting the latter to display a stronger dependency of the density of variants on gene content as they were exposed to the action of purifying natural selection for longer times and in large sectors of the population. Our results went in the opposite direction (S3 Table). From a demographic perspective, the depletion of variants in gene-regions in recent and terminal branches is at odds with a rapid population growth. In fact, the rapid radiations in some branches of Figs 1 and 2, also documented in many studies (e.g. E-M2 in Africa [47], R1a in western Eurasia [48, 49] and R1b-M269 in Europe [50, 51]), lead to the expectation of a pattern mimicking relaxed or null selection [52, 53].

We then conclude that purifying selection, if any, was not a force strong enough to severely bias the representation of variation among our chromosomal regions as produced by the mutational process.

### Single nucleotide mutations in their context

Our dataset displayed remarkable differences in the pattern of variants observed at non-CpG and CpG sites, in agreement with consolidated observations for the rest of the genome [19, 20]. As far as CpG's are concerned, a rate 12.3 fold higher than in non-CpG dinucleotides was obtained among 4,933 *de novo* events (Table 2 in ref. [13]), a value similar to that obtained from 38 million of fixed differences among three primate species [54]. Our results then support the idea that this increase quantitatively impacts the MSY in the same way as autosomes.

We show that at least part of the regional variation in the occurrence of mutations and density of variable sites in the MSY is related to gene, GC and simple repeat content [22, 55] and replication timing [38, 39] with correlations similar to the rest of the genome. As some of the features here considered refer to the entirety of each bin, we conclude that their influx on the fragments directly analysed here is exerted through context effects [54, 56].

We could not test the co-occurrence of closely spaced mutations [21, 23] as the physical distance between our variants is influenced by how our 5,274 fragments are interspersed by non-sequenced (mostly repetitive) DNA stretches. Nevertheless some suggestive examples that this phenomenon could be captured in the phylogeny exist, as we have 31 instances of mutations at <100 bp from each other in the same branch (also in short ones as branch n. 93).

In summary, we demonstrate that in a nuclear system devoid of recombination the pattern of accumulation of variants follows the same fundamental patterns observed in the autosomes for standing and *de novo* variation. We then conclude that the array of variants reflects almost directly the modes of the mutational process, which in turn results from the overlay of replication-dependent and-independent mechanisms whose final outcome is a nucleotide substitution [19].The joint effects of the genome features here examined, plus other yet unidentified ones, result in a strong regional heterogeneity in the density of variants.Under these circumstances a straightforward conclusion is that a given figure for the substitution rate only makes sense if bound to a specific DNA region. Hence, the transfer of an autosomal rate to the MSY (with the due adjustments) can be considered an authorized procedure [4, 11], but only as a first approximation, as far as the autosomal rate results from a summation over genomic regions not necessarily equally represented on the MSY. For example, the highly mutable CpG dinucleotides amount to 1.08% in our regions as compared to the autosomal 1.85% [13].

### Narrowing onto a specific regional rate

In a previous study, we adopted a figure of 0.64 × 10^−9^/site/year (equivalent to one event every 1,045 years over our amount of sequence) for the "basal" substitution rate in our 1.5 Mb region, obtained by transposing the *de novo* autosomal rate. This procedure needed to take into account the male:female mutation ratio. As a matter of fact the above value, coincident with that by Mendez et al. [11], remained in the lower half of the range currently proposed (0.53–1.0 × 10^−9^) [57].

By putting two ancient specimens sequenced at high coverage in the context of 104 present-day chromosomes representative of a large portion of extant diversity we now revise our estimate and propose a substitution rate of 0.716 × 10^−9^/site/year (95% interval 0.619–0.815) to be specifically used for the 1.5Mb region here examined. We observe that this value, too, is lower than any of those recently estimated on deeply rooted pedigrees [18] for four large regions of the MSY, one of which (XDG, X-degenerate region) embracing most of our sequences. We consider this residual discrepancy worth of further investigation but not surprising for at least two reasons. First, the regions here examined are not the same as in Helgason et al.'s study, and thus it is not unexpected that differences can emerge in the estimated mutation rate between the two studies. In this context, internal regional heterogeneity of rates at the same scale here examined can hardly be tested in pedigrees, due to paucity of mutational events. Second, the observation of a reduction of the evolutionary effective rate as compared to the pedigree-based one is well established for both uniparentally inherited portions of the genome [58, 59].

Additionally, we observed a remarkable heterogeneity in the distribution of variants, not only across different regions, but also across lineages and different times. Hg A00 stands out for showing strong associations with almost all genomic features considered. In the rest of the tree Hg's A0, A1a, A2'3 and B differ from Hg's DE, FC and R, and ancient branches differ from recent ones. The two levels are not entirely independent, as far as recent branches are enriched in lineages belonging to Hg's DE and R. It is possible that different social habits, lifestyles and environmental conditions experienced by populations harbouring different haplogroups resulted in systematic variations of the generation time and average paternal age at conception [8]. These have been suggested to affect differentially replication-dependent and non-dependent mutations, altering their relative proportions [9, 19].

### Implications of the region specific rate for recent human evolution

The rate reported above resulted in revised values for the estimated dates of nodes relevant to the genetic history in and outside Africa (Fig 2). For most directly comparable nodes our dates displayed a very good general agreement with those obtained in a recent report which also used an evolutionary-based rate for the entire XDG [8]. It is worth stressing that these authors used a figure of 0.74 × 10^−9^/site/year obtained from the regions of overlap with genomic sequences of two ancient specimens other than those examined here.

In particular, the two deepest nodes of the tree highlight the survival, in Central-Western Africa of lineages whose coalescence (291 ky, 95% C.I. 253–343) predates the emergence of anatomically modern features in the fossil record. Two nodes informative to set the lower bound for the exit Out of Africa are dated 69 kya (DE, 95% C.I. 59–79) and 72 kya (CF, 95% C.I. 63–83), somewhat converging towards dates derived from the mtDNA [60]. The Ust'-Ishim specimen is 4 mutations (7690182, 15272472, 18787745 and 19036418) downstream to the node basal to Hg K(xLT). As one of these mutations is shared with the N subject NA18558, Ust'-Ishim lies onto the NO lineage. These two lineages coalesce at 48.3 kya (95% C.I. 45.6–52.3). The assignment of the Loschbour to the I2-P37.2 branch is confirmed [28], with 8 private variants, and a coalescence with the other I2-P37 chromosome examined here at 13 kya (95% C.I. 9.8–16.0).

Overall, nodes basal to lineages which showed rapid radiations in Europe, reminiscent of a fast population growth, display dates closely matching the alleged introduction of the Neolithic cultural package [61]. The node basal to the starlike radiation involving a subset of R1b1-M269 chromosomes is estimated at 7.2 kya (95% C.I. 5.7–9.0). Two I2-M26 chromosomes coalesce at 7.0 kya, (95% C.I. 4.2–10.5) a date in line in an archaeologically documented expansion in Sardinia, where this lineage is prevalent [6]. The node basal to R1a is estimated at 8.5 kya (95% C.I. 6.2–11.1), with a date of 6.7 kya (95% C.I. 4.6–8.9) for the European branch and an age of 3.8 kya (95% C.I. 2.3–5.8) for the M458 subclade. This interval is compatible with the period of the massive migration from the Steppe-Pontic region [62, 63] to Eastern Europe, where M458 is mainly distributed today [49].

## Conclusion

The present study reveals that knowledge on the regional variation in the propensity to accumulate mutations should become a prerequisite in any study aimed at genetic dating of the MSY phylogeny. The evolutionary-based rate worked out here served to improve accuracy in the estimation of central points of node ages. Our results may imply that rates obtained as averages over large genomic regions perhaps have smaller confidence intervals, but not necessarily centered on the value appropriate for a particular subregion. We look forward for a finer modulation and the use of varied rates instead of the simple scaling-up or down of a single figure for all chromosomal regions and sites.

## Supporting Information

S1 Fig Results of dating for phylogenetic trees obtained with variable positions located in bins 3–9 (A) and 10–14 (B) with an unique substitution rate (0.716 × 10^−9^/site/year). Ages are in ky before present. CI are represented as blue bars.
(PDF)

S2 Fig Occurrence of mutations at positions within CpG dinucleotides as a function of occurrence of mutations at non-CpG positions over the whole tree in 18 out of 19 Y-chromosomal bins. (PPTX)

S3 Fig Occurrence of mutations per 100 kb of sequenced DNA in 18 out of 19 Y-chromosomal bins as a function of occupancy by genes (A), genic transcribed regions (B), EST's (C), GC content of the whole bin (D), simple repeat content in the bin (E) and average replication time score (see Materials and Methods); open squares = cell line BG01, filled squares = cell line BG02, the two regression lines closely overlap (F). (PPTX)

S4 Fig Comparison between observed values (dots) of the density of variants in 19 bins and predicted values (solid lines) based on multiple regression against 7 variables describing genomic features. (PPTX)

S1 Table List of individuals entered in the study and their haplogroup assignment (in the same order of Fig 1, top to bottom). (supplied as a worksheet).
(XLSX)

S2 Table List of mutations and their assignment to the branches of the tree depicted in Fig 1. (supplied as a worksheet).
(XLSX)

S3 Table Parameters of the correlations between measures of genomic features (rows) and number of mutations/100 kb in different subsections of the tree (columns). (supplied as a worksheet).
(XLSX)

S1 Text Noteworthy features in the tree of Fig 1. (DOCX)

S2 Text Main discrepancies in the tree chronology when using genomic subregions with high and low accumulation of variants. (DOCX)

S3 Text Correlations between number of variants and genomic features of 18 sequence bins across 8 main haplogroups. (DOCX)

## References

[1] Rito T, Richards MB, Fernandes V, Alshamali F, Cerny V, Pereira L, et al The first modern human dispersals across Africa. PLoS ONE. 2013;8:e80031 10.1371/journal.pone.0080031 24236171 PMC3827445

[2] Underhill PA, Shen P, Lin AA, Jin L, Passarino G, Yang WH, et al Y chromosome sequence variation and the history of human populations. Nat Genet. 2000;26:358–61. 11062480 10.1038/81685

[3] Poznik GD, Henn BM, Yee M-C, Sliwerska E, Euskirchen GM, Lin AA, et al Sequencing Y chromosomes resolves discrepancy in time to common ancestor of males versus females. Science. 2013;341:562–5. 10.1126/science.1237619 23908239 PMC4032117

[4] Scozzari R, Massaia A, Trombetta B, Bellusci G, Myres NM, Novelletto A, et al An unbiased resource of novel SNP markers provides a new chronology for the human Y chromosome and reveals a deep phylogenetic structure in Africa. Genome Res. 2014;24:535–44. 10.1101/gr.160788.113 24395829 PMC3941117

[5] Wei W, Ayub Q, Chen Y, McCarthy S, Hou Y, Carbone I, et al A calibrated human Y-chromosomal phylogeny based on resequencing. Genome Res. 2013;23:388–95. 10.1101/gr.143198.112 23038768 PMC3561879

[6] Francalacci P, Morelli L, Angius A, Berutti R, Reinier F, Atzeni R, et al Low-pass DNA sequencing of 1200 Sardinians reconstructs European Y-chromosome phylogeny. Science. 2013;341:565–9. 10.1126/science.1237947 23908240 PMC5500864

[7] Lippold S, Xu H, Ko A, Li M, Renaud G, Butthof A, et al Human paternal and maternal demographic histories: insights from high-resolution Y chromosome and mtDNA sequences. Investig Genet. 2014;5:13 10.1186/2041-2223-5-13 25254093 PMC4174254

[8] Karmin M, Saag L, Vicente M, Wilson Sayres MA, Järve M, Talas UG, et al A recent bottleneck of Y chromosome diversity coincides with a global change in culture. Genome Res. 2015;25:459–66. 10.1101/gr.186684.114 25770088 PMC4381518

[9] Hallast P, Batini C, Zadik D, Maisano Delser P, Wetton J, Arroyo-Pardo E, et al The Y-chromosome tree bursts into leaf: 13,000 high confidence SNPs covering the majority of known clades. Mol Biol Evol. 2015;32:661–73. 10.1093/molbev/msu327 25468874 PMC4327154

[10] Batini C, Hallast P, Zadik D, Delser PM, Benazzo A, Ghirotto S, et al Large-scale recent expansion of European patrilineages shown by population resequencing. Nat Commun. 2015;6. 10.1038/ncomms8152 PMC4441248 25988751

[11] Mendez FL, Krahn T, Schrack B, Krahn A-M, Veeramah KR, Woerner AE, et al An African American paternal lineage adds an extremely ancient root to the human Y chromosome phylogenetic tree. Am J Hum Genet. 2013;92:454–9. 10.1016/j.ajhg.2013.02.002 23453668 PMC3591855

[12] Awadalla P, Gauthier J, Myers RA, Casals F, Hamdan FF, Griffing AR, et al Direct measure of the de novo mutation rate in autism and schizophrenia cohorts. Am J Hum Genet. 2010;87:316–24. 10.1016/j.ajhg.2010.07.019 20797689 PMC2933353

[13] Kong A, Frigge ML, Masson G, Besenbacher S, Sulem P, Magnusson G, et al Rate of *de novo* mutations and the importance of father's age to disease risk. Nature. 2012;488:471–5. 10.1038/nature11396 22914163 PMC3548427

[14] Roach JC, Glusman G, Smit AF, Huff CD, Hubley R, Shannon PT, et al Analysis of genetic inheritance in a family quartet by whole-genome sequencing. Science. 2010;328:636–9. 10.1126/science.1186802 20220176 PMC3037280

[15] Elhaik E, Tatarinova TV, Klyosov AA, Graur D. The ‘extremely ancient’ chromosome that isn’t: a forensic bioinformatic investigation of Albert Perry’s X-degenerate portion of the Y chromosome. Eur J Hum Genet. 2014:1–6. 10.1038/ejhg.2013.303 PMC4135414 24448544

[16] Mendez FL, Veeramah KR, Thomas MG, Karafet T, Hammer MF. Reply to 'The'extremely ancient' chromosomome that ins't'. Eur J Hum Genet. 2014. 10.1038/ejhg.2014.148 PMC4402626 25315660

[17] Elhaik E, Tatarinova TV, Klyosov AA, Graur D. Reply to Mendez et al: the `extremely ancient' chromosome that still isn't. Eur J Hum Genet. 2015;23:567–8. 10.1038/ejhg.2014.227 25315661 PMC4402642

[18] Helgason A, Einarsson AW, Guðmundsdóttir VB, Sigurðsson Á, Gunnarsdóttir ED, Jagadeesan A, et al The Y-chromosome point mutation rate in humans. Nat Genet. 2015:Epub ahead of print. 10.1038/ng.3171 25807285

[19] Ségurel L, Wyman MJ, Przeworski M. Determinants of mutation rate variation in the human germline. Annu Rev Genomics Hum Genet. 2014;15:47–70. 10.1146/annurev-genom-031714-125740 25000986

[20] Makova KD, Hardison RC. The effects of chromatin organization on variation in mutation rates in the genome. Nat Rev Genet. 2015;16:213–23. 10.1038/nrg3890 25732611 PMC4500049

[21] Michaelson JJ, Shi Y, Gujral M, Zheng H, Malhotra D, Jin X, et al Whole-genome sequencing in autism identifies hot spots for de novo germline mutation. Cell. 2012;151:1431–42. 10.1016/j.cell.2012.11.019 23260136 PMC3712641

[22] Hellmann I, Prüfer K, Ji H, Zody MC, Paabo S, Ptak SE. Why do human diversity levels vary at a megabase scale? Genome Res. 2005;15:1222–31. 16140990 10.1101/gr.3461105 PMC1199536

[23] Francioli LC, Polak PP, Koren A, Menelaou A, Chun S, Renkens I, et al Genome-wide patterns and properties of *de novo* mutations in humans. Nat Genet. 2015. 10.1038/ng.3292 PMC4485564 25985141

[24] Hughes JF, Skaletsky H, Pyntikova T, Graves TA, van Daalen SKM, Minx PJ, et al Chimpanzee and human Y chromosomes are remarkably divergent in structure and gene content. Nature. 2010;463:536–9. 10.1038/nature08700 20072128 PMC3653425

[25] Wolfe KH, Sharp PM, Li W-H. Mutation rates differ among regions of the mammalian genome. Nature. 1989;337:283–5. 2911369 10.1038/337283a0

[26] O'Fallon B. Purifying selection causes widespread distortions of genealogical structure on the human X chromosome. Genetics. 2013;194:485–92. 10.1534/genetics.113.152074 23589459 PMC3664857

[27] Fu Q, Li H, Moorjani P, Jay F, Slepchenko SM, Bondarev AA, et al Genome sequence of a 45,000-year-old modern human from western Siberia. Nature. 2014;514:445–9. 10.1038/nature13810 25341783 PMC4753769

[28] Lazaridis I, Patterson N, Mittnik A, Renaud G, Mallick S, Kirsanow K, et al Ancient human genomes suggest three ancestral populations for present-day Europeans. Nature. 2014;513:409–13. 10.1038/nature13673 25230663 PMC4170574

[29] Tamura K, Peterson D, Peterson N, Stecher G, Nei M, Kumar S. MEGA5: Molecular Evolutionary Genetics Analysis using maximum likelihood, evolutionary distance, and maximum parsimony methods. Mol Biol Evol. 2011;28:2731–9. 10.1093/molbev/msr121 21546353 PMC3203626

[30] Bandelt HJ, Forster P, Rohl A. Median-joining networks for inferring intraspecific phylogenies. Mol Biol Evol. 1999;16:37–48. 10331250 10.1093/oxfordjournals.molbev.a026036

[31] Drummond AJ, Rambaut A. BEAST: Bayesian evolutionary analysis by sampling trees. BMC Evol Biol 2007;7:214 17996036 10.1186/1471-2148-7-214 PMC2247476

[32] Hamilton MJ, Burger O, DeLong JP, Walker RS, Moses ME, Brown JH. Population stability, cooperation, and the invasibility of the human species. Proc Natl Acad Sci USA. 2009;106:12255–60. 10.1073/pnas.0905708106 19592508 PMC2718330

[33] Ammerman AJ, Cavalli-Sforza LL. The Neolithic transition and the genetics of populations in Europe Princeton: Princeton University Press 1984.

[34] Boone JL. Subsistence strategies and early human population history: an evolutionary ecological perspective World Archaeol. 2002;34:6–25. 16475305 10.1080/00438240220134232

[35] Weddington N, Stuy A, Hiratani I, Ryba T, Yokochi T, Gilbert DM. ReplicationDomain: a visualization tool and comparative database for genome-wide replication timing data. BMC Bioinformatics. 2008;9:530 10.1186/1471-2105-9-530 19077204 PMC2636809

[36] Ross MT, Grafham DV, Coffey AJ, Scherer S, McLay K, Muzny D, et al The DNA sequence of the human X chromosome. Nature. 2005;434:325–37. 15772651 10.1038/nature03440 PMC2665286

[37] Lahn BT, Page DC. Four evolutionary strata on the human X chromosome. Science. 1999;286:964–7. 10542153 10.1126/science.286.5441.964

[38] Koren A, Polak P, Nemesh J, Michaelson JJ, Sebat J, Sunyaev SR, et al Differential relationship of DNA replication timing to different forms of human mutation and variation. Am J Hum Genet. 2012;91:1033–40. 10.1016/j.ajhg.2012.10.018 23176822 PMC3516607

[39] Stamatoyannopoulos JA, Adzhubei I, Thurman RE, Kryukov GV, Mirkin SM, Sunyaev SR. Human mutation rate associated with DNA replication timing. Nat Genet. 2009;41:393–5. 10.1038/ng.363 19287383 PMC2914101

[40] Niederstätter H, Berger B, Erhart D, Willuweit S, Geppert M, Gassner C, et al Multiple recurrent mutations at four human Y-chromosomal single nucleotide polymorphism sites in a 37 bp sequence tract on the ARSDP1 pseudogene. Forensic Sci Intl: Genetics. 2013;7:593–600. 10.1016/j.fsigen.2013.05.010 23810651

[41] Rosser ZH, Balaresque P, Jobling MA. Gene conversion between the X chromosome and the male-specific region of the Y chromosome at a translocation hotspot. Am J Hum Genet 2009;85:130–4. 10.1016/j.ajhg.2009.06.009 19576564 PMC2706966

[42] Cruciani F, Trombetta B, Macaulay V, Scozzari R. About the X-to-Y gene conversion rate. Am J Hum Genet. 2011;86:495–7. 10.1016/j.ajhg.2010.01.033 PMC2833382 20215010

[43] Trombetta B, Cruciani F, Underhill PA, Sellitto D, Scozzari R. Footprints of X-to-Y gene conversion in recent human evolution. Mol Biol Evol. 2010;27:714–25. 10.1093/molbev/msp231 19812029

[44] Trombetta B, Sellitto D, Scozzari R, Cruciani F. Inter- and intraspecies phylogenetic analyses reveal extensive X–Y gene conversion in the evolution of gametologous sequences of human sex chromosomes. Mol Biol Evol. 2014;31:2108–23. 10.1093/molbev/msu155 24817545 PMC4104316

[45] Rozen S, Marszalek JD, Alagappan RK, Skaletsky H, Page DC. Remarkably little variation in proteins encoded by the Y chromosome's single-copy genes, implying effective purifying selection. Am J Hum Genet. 2009;85:923–8. 10.1016/j.ajhg.2009.11.011 20004767 PMC2790577

[46] Wilson Sayres MA, Lohmueller KE, Nielsen R. Natural selection reduced diversity on human Y chromosomes. PLoS Genet. 2014;10:e1004064 10.1371/journal.pgen.1004064 24415951 PMC3886894

[47] Montano V, Ferri G, Marcari V, Batini C, Anyaele O, Destro-Bisol G, et al The Bantu expansion revisited: a new analysis of Y chromosome variation in Central Western Africa. Mol Ecol. 2011;20:2693–708. 10.1111/j.1365-294X.2011.05130.x 21627702

[48] Underhill P, Poznik GD, Rootsi S, Järve M, Lin A, Wang J, et al The phylogenetic and geographic structure of Y-chromosome haplogroup R1a. Eur J Hum Genet. 2014;23:124–31. 10.1038/ejhg.2014.50 24667786 PMC4266736

[49] Underhill PA, Myres NM, Rootsi S, Metspalu M, Zhivotovsky LA, King RJ, et al Separating the post-Glacial coancestry of European and Asian Y chromosomes within haplogroup R1a. Eur J Hum Genet. 2010;18:479–84. 10.1038/ejhg.2009.194 19888303 PMC2987245

[50] Balaresque P, Bowden GR, Adams SM, Leung H-Y, King TE, Rosser ZH, et al A predominantly Neolithic origin for European paternal lineages. PLoS Biol. 2010;8:e1000285 10.1371/journal.pbio.1000285 20087410 PMC2799514

[51] Myres NM, Rootsi S, Lin AA, Järve M, King RJ, Kutuev I, et al A major Y-chromosome haplogroup R1b Holocene era founder effect in Central and Western Europe. Eur J Hum Genet. 2011;19:95–101. 10.1038/ejhg.2010.146 20736979 PMC3039512

[52] Ralph P, Coop G. The geography of recent genetic ancestry across Europe. PLoS Biol. 2013;11:e1001555 10.1371/journal.pbio.1001555 23667324 PMC3646727

[53] Keinan A, Clark AG. Recent explosive human population growth has resulted in an excess of rare genetic variants. Science. 2012;336:740–3. 10.1126/science.1217283 22582263 PMC3586590

[54] Elango N, Kim S-H, Vigoda E, Yi SV, Nisc Comparative Sequencing Program. Mutations of different molecular origins exhibit contrasting patterns of regional substitution rate variation. PLoS Comput Biol. 2008;4:e1000015 10.1371/journal.pcbi.1000015 18463707 PMC2265638

[55] Schaibley VM, Zawistowski M, Wegmann D, Ehm MG, Nelson MR, St Jean PL, et al The influence of genomic context on mutation patterns in the human genome inferred from rare variants. Genome Res. 2013;23:1974–84. 10.1101/gr.154971.113 23990608 PMC3847768

[56] Hodgkinson A, Ladoukakis E, Eyre-Walker A. Cryptic variation in the human mutation rate. PLoS Biology. 2009;7:e27. 10.1371/journal.pbio.1000027 PMC2634788 19192947

[57] Wang CC, Gilbert MTP, Jin L, Li H. Evaluating the Y chromosomal timescale in human demographic and lineage dating. Investig Genet. 2014;5:12 10.1186/2041-2223-5-12 25215184 PMC4160915

[58] Henn BM, Gignoux CR, Feldman MW, Mountain JL. Characterizing the time dependency of human mitochondrial DNA mutation rate estimates. Mol Biol Evol. 2009;26:217–30. 10.1093/molbev/msn244 18984905

[59] Zhivotovsky LA, Underhill PA, Feldman MW. Difference between evolutionarily effective and germ-line mutation rate due to stochastically varying haplogroup size. Mol Biol Evol. 2006;23:2268–70. 16956974 10.1093/molbev/msl105

[60] Soares P, Alshamali F, Pereira JB, Fernandes V, Silva NM, Afonso C, et al The expansion of mtDNA haplogroup L3 within and out of Africa. Mol Biol Evol. 2011;29:915–27. 10.1093/molbev/msr245 22096215

[61] Rowley-Conwy P. Westward Ho! Curr Anthrop. 2011;52:S431–S51.

[62] Allentoft ME, Sikora M, Sjogren K-G, Rasmussen S, Rasmussen M, Stenderup J, et al Population genomics of Bronze Age Eurasia. Nature. 2015;522:167–72. 10.1038/nature14507 26062507

[63] Haak W, Lazaridis I, Patterson N, Rohland N, Mallick S, Llamas B, et al Massive migration from the steppe was a source for Indo-European languages in Europe. Nature. 2015;522:207–11. 10.1038/nature14317 25731166 PMC5048219

[64] van Oven M, Van Geystelen A, Kayser M, Decorte R, Lermuseau MH. Seeing the wood for the trees: a minimal reference phylogeny for the human Y chromosome. Hum Mut. 2014;35:187–91. 10.1002/humu.22468 24166809

[65] Trombetta B, D’Atanasio E, Massaia A, Ippoliti M, Coppa A, Candilio F, et al Phylogeographic refinement and large scale genotyping of human Y chromosome haplogroup E provide new insights into the dispersal of early pastoralists in the African continent. Genome Biol Evol. 2015:6 24. pii: evv118. [Epub ahead of print]. 10.1093/gbe/evv118 PMC4524485 26108492

