# Supplementary figures and images for: Regional Differences in the Accumulation of SNPs on the Male-Specific Portion of the Human Y Chromosome Replicate Autosomal Patterns: Implications for Genetic Dating
Source: PLoS One. 2015 Jul 30;10(7):e0134646. doi: 10.1371/journal.pone.0134646 (PMC4520482; doi:10.1371/journal.pone.0134646)

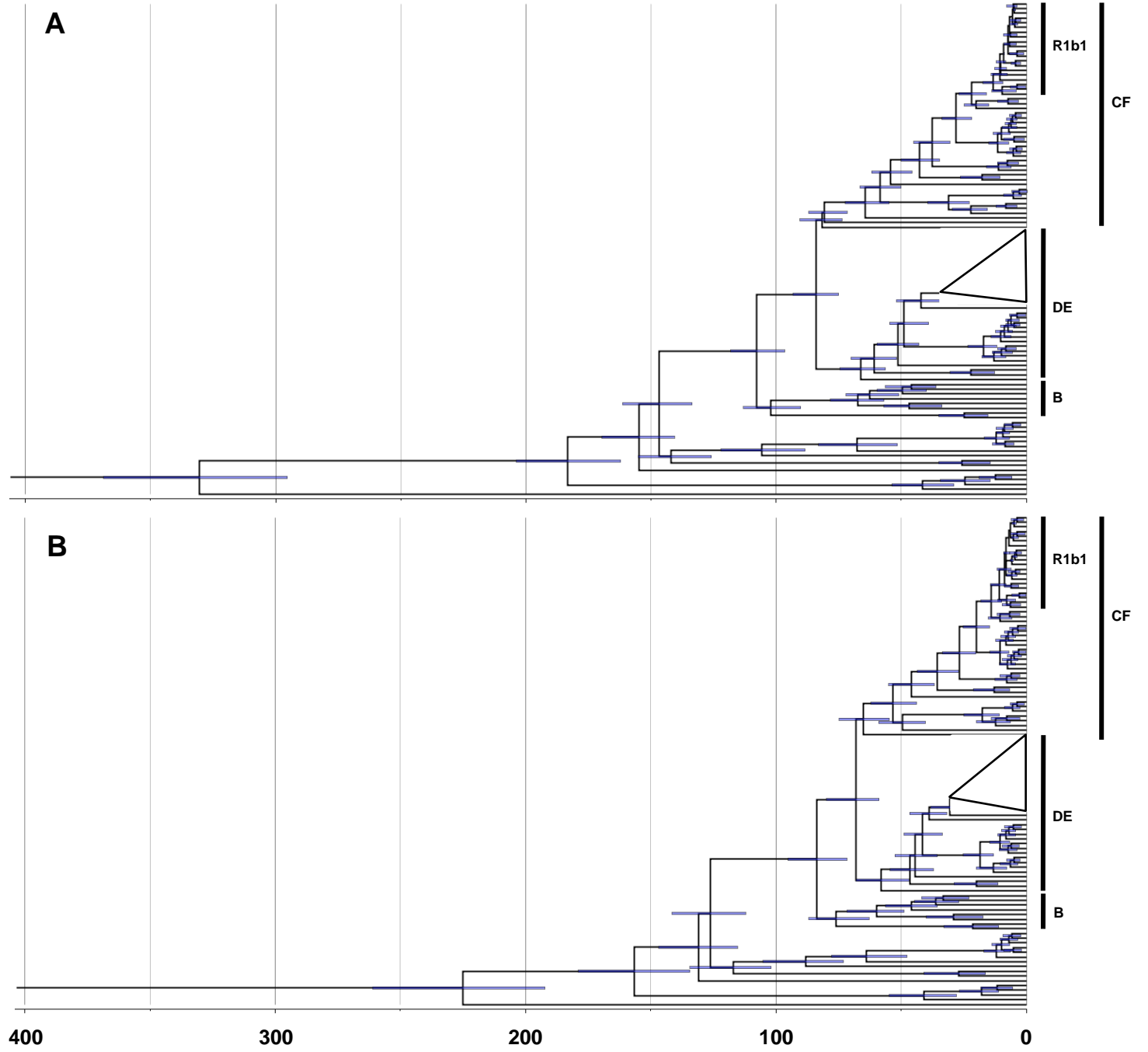

Supplement: S1 Fig — Ages are in ky before present. CI are represented as blue bars. (PDF) [file pone.0134646.s001.pdf]

## Slide 1
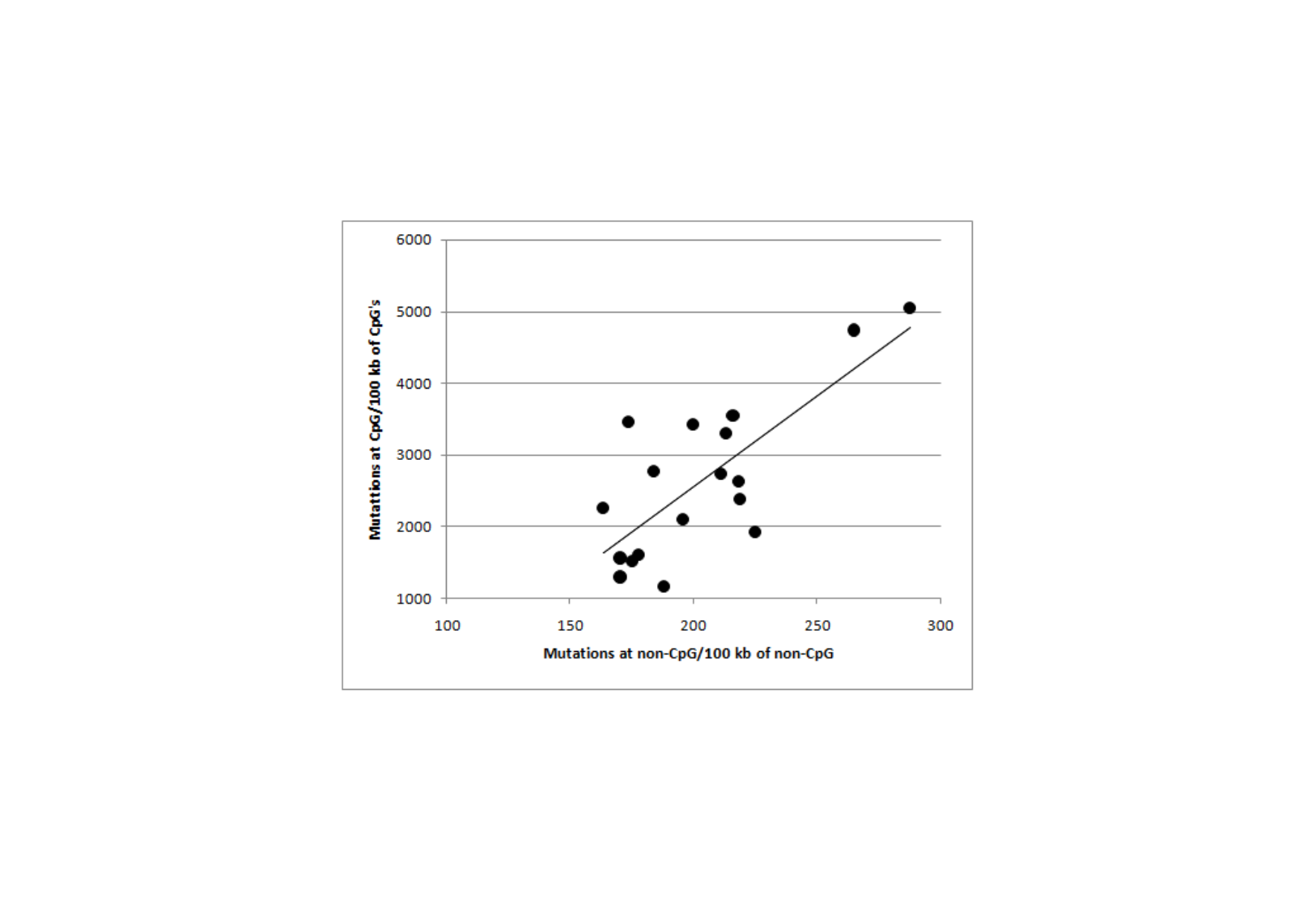

Supplement: S2 Fig — (PPTX) [file pone.0134646.s002.pptx]

## Slide 1
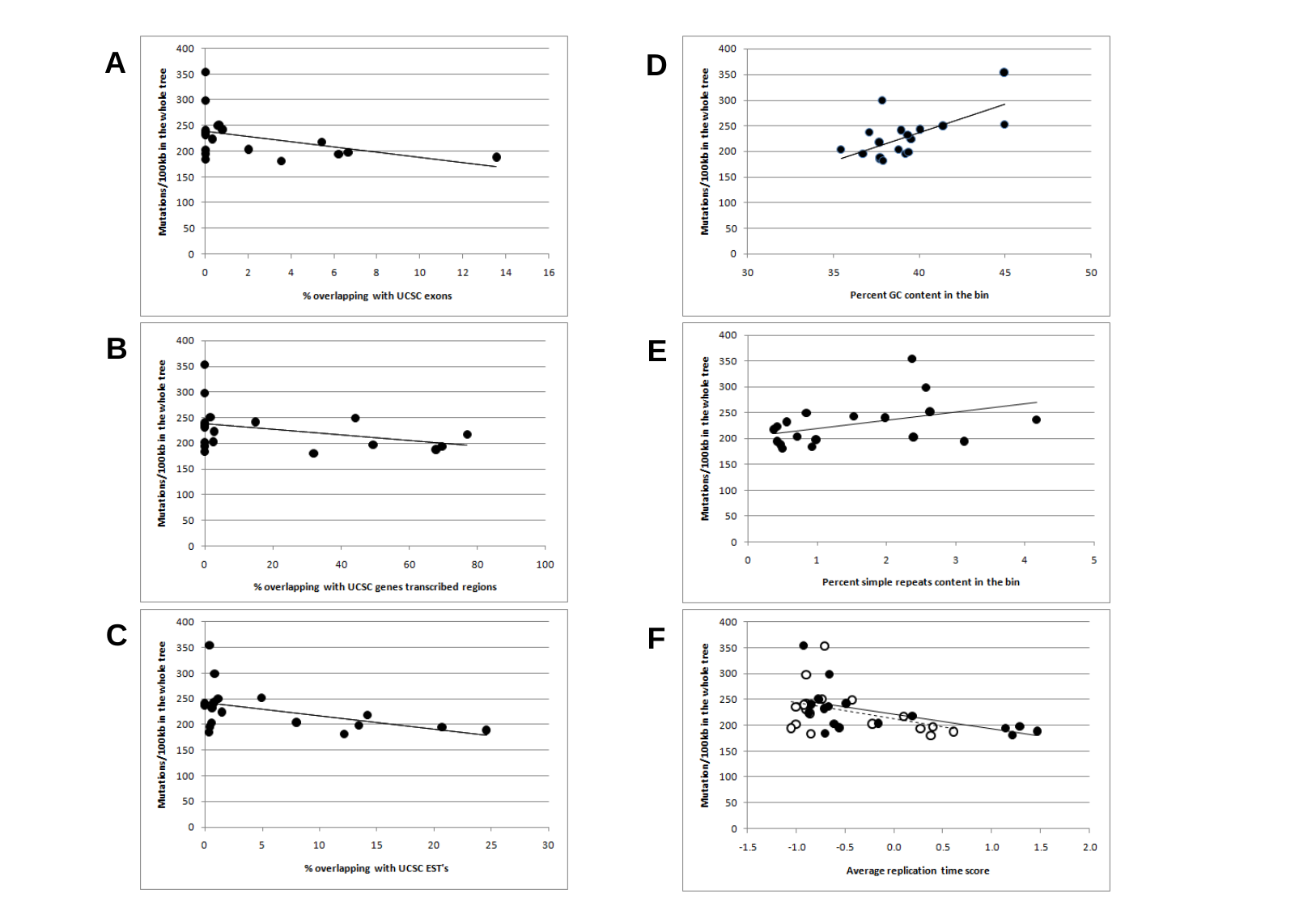

A
D
B
E
C
F

Supplement: S3 Fig — (PPTX) [file pone.0134646.s003.pptx]

## Slide 1
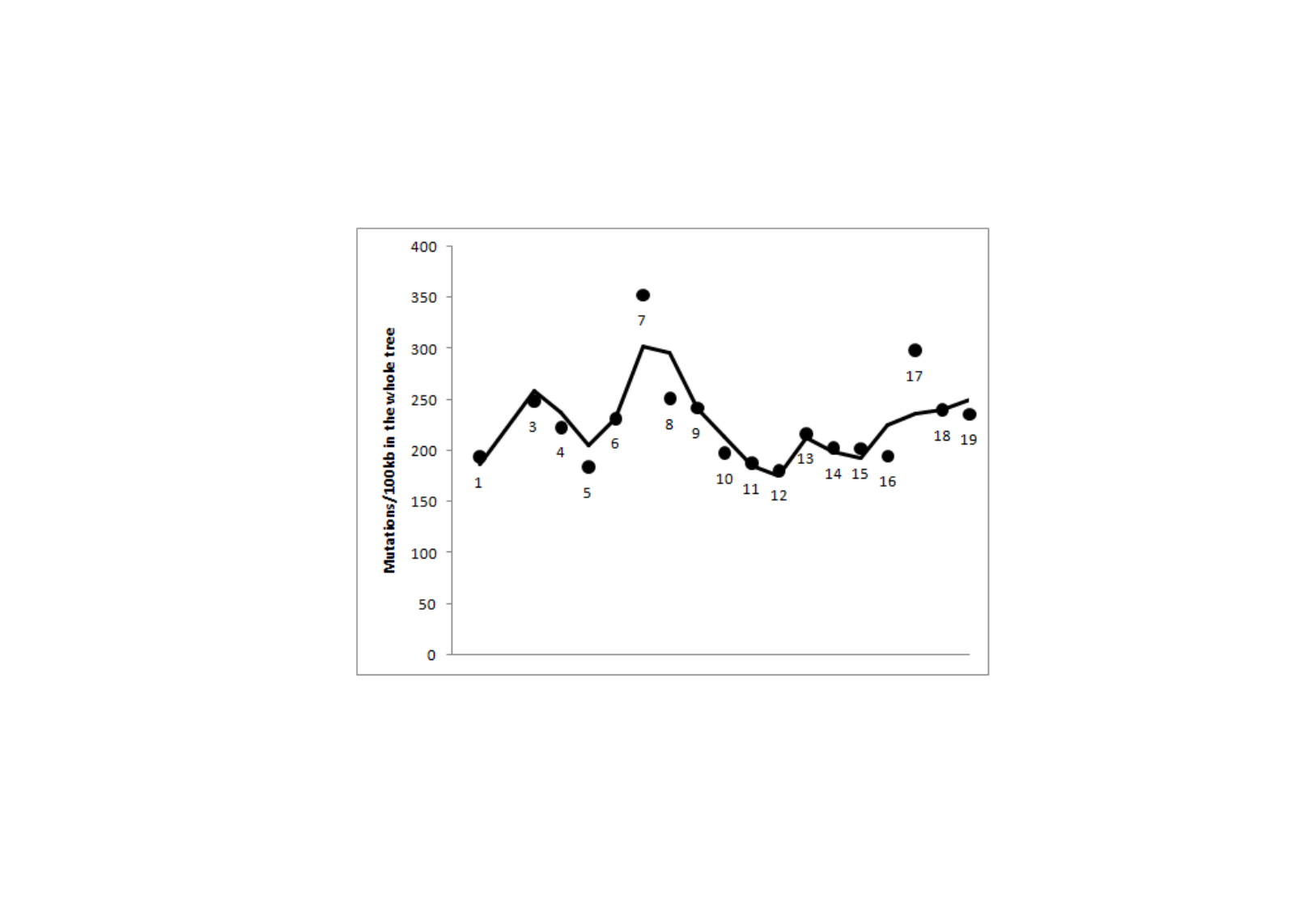

Supplement: S4 Fig — (PPTX) [file pone.0134646.s004.pptx]
