# Supplementary material for: Regional Differences in the Accumulation of SNPs on the Male-Specific Portion of the Human Y Chromosome Replicate Autosomal Patterns: Implications for Genetic Dating
Source: PLoS One. 2015 Jul 30;10(7):e0134646. doi: 10.1371/journal.pone.0134646 (PMC4520482; doi:10.1371/journal.pone.0134646)
Supplement: S1 Text — (DOCX) [file pone.0134646.s008.docx]

Supplemental Text 1.

Noteworthy features in the tree of Figure 1 (see S1 Table for detailed individual genotypes).

- The A00 specimen defines a branch far deeper than any other branch in agreement previous reports (Karmin et al., 2015; Mendez et al., 2013), with 393 variants that separate it from the node basal to all other haplogroups;
- within A3, two new samples (S76 and S77) add a new A3-M13 subclade;
- a new sample (S73), representative of the rare Hg A3-M28, reveals that Hg A3 is remarkably deeply rooted;
- an additional sample (S75) within Hg A2 confirms (Poznik et al., 2013) the relatively deep coalescence of A2 lineages sampled in central vs southern Africa;
- within Hg B2b, a new lineage sampled in Central Africa (S74) splits a branch previously thought to be basal to the entire Hg;
- within Hg E, the rare eastern African Hg E1b1a-M329 (S21) is confirmed to be a sister clade of the common sub-Saharan Hg E1b1a-M2, but with a large number (60) of lineage-specific mutations;
- within R1a the chromosomes sampled in Asia and Europe cluster separately, replicating the partition previously observed (Karmin et al., 2015; Underhill et al., 2014), and a distinctive branch groups together three R1a-M458 subjects (S59-S61) in the European clade;
- within R1b, two subjects carrying the derived allele at V88 (S42 and S70)(Cruciani et al., 2010) plus an additional one (S33) previously classified as paragroup R1b-M343*(xP297, V88, M335) form a multifurcation together with the branch leading to R1b1-M269;
- within R1b1-M269 two branches (152 and 153 in Figure 1) defined by 7 and 3 mutations respectively, split a single paragroup so far described as R1b1-M269*(xL23);

REFERENCES FOR S1 TEXT

Cruciani F, Trombetta B, Sellitto D, Massaia A, Destro-Bisol G, Watson E, Beraud Colomb E, Dugoujon JM, Moral P, Scozzari R. 2010. Human Y chromosome haplogroup R-V88: a paternal genetic record of early mid Holocene trans-Saharan connections and the spread of Chadic languages. Eur J Hum Genet 18:800-807 and Corrigendum 807.

Karmin M, Saag L, Vicente M, Wilson Sayres MA, Järve M, Talas UG, Rootsi S, Ilumäe A-M, Mägi R, Mitt M, Pagani L, Puurand T, et al. 2015. A recent bottleneck of Y chromosome diversity coincides with a global change in culture. Genome Res 25:459-466.

Mendez FL, Krahn T, Schrack B, Krahn A-M, Veeramah KR, Woerner AE, Fomine FLM, Bradman N, Thomas MG, Karafet TM, Hammer MF. 2013. An African American paternal lineage adds an extremely ancient root to the human Y chromosome phylogenetic tree. Am J Hum Genet 92:454-459.

Poznik GD, Henn BM, Yee M-C, Sliwerska E, Euskirchen GM, Lin AA, Snyder M, Quintana-Murci L, Kidd JM, Underhill PA, Bustamante CD. 2013. Sequencing Y chromosomes resolves discrepancy in time to common ancestor of males versus females. Science 341:562-565.

Underhill P, Poznik GD, Rootsi S, Järve M, Lin A, Wang J, Passarelli B, Kanbar J, Myres N, King R, Di Cristofaro J, Sahakyan H, et al. 2014. The phylogenetic and geographic structure of Y-chromosome haplogroup R1a. Eur J Hum Genet 23:124-131.
