# Supplementary material for: Regional Differences in the Accumulation of SNPs on the Male-Specific Portion of the Human Y Chromosome Replicate Autosomal Patterns: Implications for Genetic Dating
Source: PLoS One. 2015 Jul 30;10(7):e0134646. doi: 10.1371/journal.pone.0134646 (PMC4520482; doi:10.1371/journal.pone.0134646)
Supplement: S2 Text — (DOCX) [file pone.0134646.s009.docx]

Supplemental text 2.

Main discrepancies in tree chronology when using genomic subregions with high and low accumulation of variants (S1 Fig.)

- The total height of the tree is 331 ky in panel A but 225 ky in panel B. We remind that the positioning of the root is the result of the Bayesian process and not of the assessment of ancestral/derived states in branch 0 based on an outgroup (e.g. the chimpanzee);
- the date for the node basal to A0'1'2'3'4 is 184 kya in panel A, but 156 ky in panel B. The first date is compatible with the presence of the MRCA of A0'1'2'3'4 in pre- anatomically modern populations;
- the node basal to Hg B is 108 kya in panel A but 83 kya in panel B;
- two nodes informative to set the lower bound for the exit Out of Africa (DE and CF) are compressed between 84.4 and 80.9 kya in panel A but between 68.1 and 65.1 in panel B;
- the node basal to the radiation within Hg R1b1-M269 is 13.6 kya in panel A but 10.8 in panel B. Only the second time estimate approaches dates compatible with a Neolithic radiation.
