# Supplementary material for: Regional Differences in the Accumulation of SNPs on the Male-Specific Portion of the Human Y Chromosome Replicate Autosomal Patterns: Implications for Genetic Dating
Source: PLoS One. 2015 Jul 30;10(7):e0134646. doi: 10.1371/journal.pone.0134646 (PMC4520482; doi:10.1371/journal.pone.0134646)
Supplement: S3 Text — (DOCX) [file pone.0134646.s010.docx]

Supplemental Text 3

Correlations between number of variants and genomic features of 18 sequence bins across 8 main haplogroups (comments to S3 Table)

- A general trend towards negative r values was obtained for all haplogroups when number of variants were regressed against occupancy by exonic and transcribed sequences of UCSC genes, and EST's, reaching significance in 5 out of 24 tests (3 variables × 8 haplogroups);
- Positive correlations were found in 15/16 tests performed for the 8 haplogroups against GC content in each entire bin and in the sequenced fragments only, with significant p values in 10 cases (p-value range = 0.048-0.0003).
- Correlation between number of variants and the occupancy by simple repeats in the whole bin produced positive r values in 7 out of 8 Hg's, reaching significance for Hg A00 (p=0.0005);
- Negative correlations were obtained in 14/16 tests involving replication time in two cell lines.
